# 苯达莫司汀联合泊马度胺及地塞米松治疗复发难治多发性骨髓瘤的疗效及安全性

**DOI:** 10.3760/cma.j.issn.0253-2727.2023.06.012

**Published:** 2023-06

**Authors:** 婧 王, 颖 姚, 京晶 商, 骁 马, 琤琤 傅, 德沛 吴, 松 金

**Affiliations:** 1 苏州弘慈血液病医院血液科，苏州 215128 Department of Hematology, Soochow Hopes Hematology Hospital, Suzhou 215128, China; 2 苏州大学附属第一医院血液科，江苏省血液研究所，苏州 215006 Department of Hematology, the First Affiliated Hospital of Soochow University, Jiangsu Institute of Hematology, Suzhou 215006, China

多发性骨髓瘤（MM）迄今缺乏根治性手段，复发难治MM（RRMM）患者接受后续治疗的有效率低、生存期短。达雷妥尤单抗、泊马度胺、卡非佐米和塞利尼索等新药的上市使中国患者获得了更好的疗效，但新药在中国RRMM患者中的临床数据不足，合并髓外病灶的患者仍然疗效欠佳[1]。泊马度胺作为第三代免疫调节剂，在第一代沙利度胺和第二代来那度胺的结构基础上进行了升级，克服了交叉耐药性，循证医学研究证明，泊马度胺可使来那度胺难治患者获益[2]–[4]。苯达莫司汀是一种氮芥类抗肿瘤药物，具有烷基化和抗代谢活性双重作用，2022年NCCN指南[5]将苯达莫司汀纳入RRMM患者的治疗选择。目前，以苯达莫司汀为主的化疗方案多用于非霍奇金淋巴瘤及慢性淋巴细胞白血病，而用于MM患者的报道较少。本研究回顾性分析了苏州大学附属第一医院分院苏州弘慈血液病医院骨髓瘤病区应用苯达莫司汀、泊马度胺、地塞米松联合化疗方案（BPD方案）治疗RRMM患者的疗效及安全性。

## 病例与方法

1. 病例：回顾性分析2020年7月1日至2021年4月1日在苏州弘慈血液病医院接受BPD方案治疗的20例RRMM患者的临床资料。所有入选患者均根据《中国多发性骨髓瘤诊治指南（2020年修订）》[6]中的诊断标准确诊为MM，所有患者均完成≥1个疗程的治疗。采集的临床指标包括：性别、年龄、国际骨髓瘤工作组（IMWG）虚弱评分、M蛋白分型、肌酐清除率、国际分期系统（ISS）、修订的ISS（R-ISS）、CD138分选后的FISH、既往治疗方案及疗效、既往耐药情况、骨髓细胞形态学、骨髓免疫分型、骨髓病理检查、全身低剂量CT检查结果等。

复发MM指既往治疗至少获得部分缓解（PR）疗效，之后再次出现疾病进展。难治MM分为原发难治和复发难治，原发难治指应用诱导治疗（4～6个疗程）并未达到微小缓解（MR）及以上疗效；RRMM指最佳疗效为MR或前一线治疗后获得MR以上疗效，但是疾病在60 d内快速进展。高危细胞遗传学定义为患者送检骨髓标本经CD138分选后FISH检出t（4;14）、t（14;16）、t（14;20）、del（17p）、1q+。

2. 治疗方案：BPD方案的具体剂量为：苯达莫司汀70 mg·m^−2^·d^−1^，第1、2天；泊马度胺4 mg/d，第1～21天；地塞米松40 mg/d，第1、8、15、22天。28 d为一个周期。

3. 疗效评估：采用《中国多发性骨髓瘤诊治指南（2020年修订）》[6]的传统疗效标准进行判断。疗效分为：严格意义的完全缓解（sCR）、完全缓解（CR）、非常好的部分缓解（VGPR）、PR、MR、疾病稳定（SD）和疾病进展（PD）。总体有效率（ORR）为sCR、CR、VGPR、PR率之和。临床获益率（clinical benefit rate，CBR）为sCR、CR、VGPR、PR、MR率之和。不良反应的分级标准参照美国常见不良反应术语评定标准5.0版（CTCAE V5.0）。

4. 随访：通过查阅患者门诊、住院病历及电话进行随访，末次随访时间为2021年11月，中位随访时间为11（7～16）个月。总生存（OS）期定义为自患者诊断MM至因各种原因死亡或末次随访的时间，无进展生存（PFS）时间定义为BPD方案治疗后首次获得MR以上血液学疗效至疾病进展或复发的时间。

5. 统计学处理：采用SPSS 25.0软件进行统计学分析，计数资料以“例数（百分比）”形式表示，计量资料以“中位数（范围）”形式表示。采用Kaplan-Meier法进行生存分析。

## 结果

1. 临床特征：20例患者中男16例（80％），女4例（20％），中位年龄57（44～71）岁。IMWG虚弱评分0～1分15例（75％），2分5例（25％）。IgG型8例（40％），IgA型2例（10％），IgD型2例（10％），κ轻链型3例（15％），λ轻链型5例（25％）。肌酐清除率>40 ml/min者16例（80.0％），肌酐清除率≤40 ml/min者4例（20％）。ISS分期Ⅰ期4例（20％），Ⅱ期5例（25％），Ⅲ期11例（55％）。R-ISS分期Ⅰ期4例（20％），Ⅱ期11例（55％），Ⅲ期5例（25％）。细胞遗传学标危10例（50％），高危10例（50％）。无髓外侵犯11例（55％），伴髓外侵犯9例（45％），其中中枢神经系统受累3例（15％）。既往经历二线治疗4例（20％），三线治疗7例（35％），四线及以上治疗9例（45％）。硼替佐米耐药19例（95％），来那度胺耐药17例（85％），硼替佐米及来那度胺双重耐药16例（80％）。

2. 疗效评估：2例患者因失访未获得疗效评估数据，18例患者完成后续疗效评估及生存分析。20例患者中位治疗3（1～9）个疗程，没有患者获得CR及以上疗效，2例获得VGPR，6例获得PR，1例获得MR，8例SD，1例PD。细胞遗传学标危组9例患者中6例疗效达到PR及以上，高危组9例患者中2例疗效达到PR及以上。伴髓外侵犯组9例患者中5例疗效达到PR及以上，其中中枢神经系统侵犯组2例PR，1例SD。非髓外侵犯组9例患者中3例疗效达到PR及以上。3例二线治疗后患者1例疗效达到PR及以上，7例三线治疗后患者中4例疗效达到PR及以上，8例四线及以上治疗后患者中3例疗效达到PR及以上。17例硼替佐米耐药患者中7例疗效达到PR及以上，16例来那度胺耐药患者中7例疗效达到PR及以上，15例双重耐药患者中6例疗效达到PR及以上。第1个疗程结束后，18例患者中6例疗效达到PR及以上，第2个疗程结束后，7例疗效达到PR及以上。

3. 生存分析：6个月OS率为63.2％，12个月OS率为21.1％，中位OS时间为7（2～14）个月。6个月PFS率为31.5％，12个月PFS率为10.5％，中位PFS时间为5（2～13）个月。截至末次随访，7例患者因疾病进展终止应用BPD方案，3例患者因非疾病相关死亡终止治疗，2例疗效评估为SD的患者自行要求更换治疗方案。

4. 安全性评估：对20例患者进行安全性分析。血液学不良反应以1～2级淋巴细胞计数降低（发生率75％）、中性粒细胞计数降低（发生率70％）较为常见，贫血、血小板减少的发生率分别为40％和35％，3～4级血液学不良反应发生率为25％。3例患者需要间断输注血制品支持治疗，3例患者因3～4级血液学不良反应延迟化疗。非血液学不良反应中以1～2级乏力最常见（发生率60％），多见于接受第1个疗程化疗的患者，可自行缓解。恶心、呕吐主要为1～2级（发生率20％），对症治疗后可缓解。7例患者出现呼吸道感染，其中2例为3级，应用抗感染治疗后好转。1例患者出现1级肝功能损伤，予保肝治疗后好转。3例患者出现一过性外周血肌酐升高。1例患者出现心房颤动，治疗后复律。未发生皮疹等过敏表现。治疗相关死亡率为0。

## 讨论

苯达莫司汀是一种氮芥类抗肿瘤药物，具有烷基化和抗代谢活性双重作用，常被用于淋巴瘤和慢性淋巴细胞白血病的治疗，在多个B细胞和T细胞淋巴瘤细胞系中表现出高细胞毒性[7]。苯达莫司汀在体外实验中显著抑制了骨髓瘤细胞增殖[8]。在国外多个前瞻和回顾性临床研究中，苯达莫司汀单药或联合地塞米松、沙利度胺、来那度胺、硼替佐米、伊沙佐米、卡非佐米等治疗RRMM患者均显示出了良好的有效性和安全性[9]–[11]。苯达莫司汀单药或联合糖皮质激素治疗RRMM的ORR为36％[12]。有研究报道苯达莫司汀联合硼替佐米及地塞米松治疗RRMM的ORR达到了60.8％，中位OS时间达25.6个月，其中38％的患者出现3～4级血小板减少，23％的患者出现3级以上感染[13]。苯达莫司汀联合伊沙佐米及地塞米松时ORR达61％，中位OS时间为23.2个月，不良反应主要为贫血、白细胞减少及恶心腹泻[14]。苯达莫司汀联合卡非佐米及地塞米松治疗二线以上RRMM的研究中[15]–[16]，ORR达52％～88％，中位OS时间达30.4～56.3个月，3级以上的血液学不良反应主要为淋巴细胞减少（29％）、中性粒细胞减少（25％）和血小板减少（22％），3级以上的非血液学不良反应主要为肺部感染。苯达莫司汀联合沙利度胺及地塞米松治疗硼替佐米、来那度胺治疗后的RRMM，ORR为37％[17]。在苯达莫司汀联合来那度胺及地塞米松治疗接受三线以上治疗RRMM的研究中，ORR为49％，1年OS率达到72％，但3～4血液学不良反应发生率高达94％[18]。在苯达莫司汀联合泊马度胺及地塞米松治疗RRMM的研究中，ORR达到61％，中位OS时间达21个月，3级以上的血液学不良反应普遍存在，但可以通过药物干预得到较好的结果[19]。荟萃分析显示含苯达莫司汀的三药方案较两药方案疗效更优，且没有增加不良反应发生率[20]。联合免疫调节剂的三药方案ORR达48％～86％，中位OS时间为10.8个月以上，中位PFS时间为13～23个月，常见不良反应为淋巴细胞减少、贫血及中性粒细胞减少。国外相关研究报道，苯达莫司汀应用剂量为每疗程60～90 mg·m^−2^·d^−1^× 2 d，更大剂量组常伴随不良反应增加及患者耐受度下降[14],[16],[18]–[19]，苯达莫司汀药物最新共识中也推荐了相似剂量[21]，本研究中的患者均接受了苯达莫司汀每疗程70 mg·m^−2^·d^−1^×2 d。苯达莫司汀药物共识中提到苯达莫司汀联合免疫调节剂类药物可能获得更好疗效[21]，且本研究中的大部分患者均明确对来那度胺耐药，因此我们选择了泊马度胺联合用药。泊马度胺常用于RRMM的治疗，包括来那度胺耐药患者。泊马度胺联合卡非佐米及地塞米松（KPD方案）在印度一项真实世界回顾性研究中的ORR达65.2％，中位PFS时间和OS时间分别为11.3和28.0个月，但感染发生率较高（26％）[22]。泊马度胺联合达雷妥尤单抗（DPD方案）治疗自体造血干细胞移植后来那度胺维持治疗复发的MM患者[23]，ORR达88.3％，中位PFS时间达18.9个月。对于多线治疗复发的RRMM，单中心的研究报道显示，DPD方案的ORR为33.3％～91.7％[24]。真实世界中泊马度胺联合塞利尼索的报道较少，Chen等[25]报道，在未服用过泊马度胺的RRMM患者中，SPD方案的ORR为58％，中位PFS和OS时间为12.3和19.0个月（STOMP 1b/2期研究）。

本研究中BPD方案治疗RRMM的ORR为44.4％，较上述研究低，考虑与本研究纳入较多（80％）三线及以上治疗的RRMM患者有关。本方案对部分患者起效快，符合多线复发患者的治疗目标，但缓解维持时间较短。亚组分析显示，细胞遗传学高危组患者疗效不佳，髓外受累尤其是中枢神经系统受累患者疗效达到PR及以上比例高于无髓外病变患者，国内有相关报道显示伴髓外病灶患者应用含苯达莫司汀的联合方案治疗后取得良好疗效[26]。故此方案可能是髓外累及的患者潜在的优选方案之一。在安全性方面，3～4级不良反应集中于血液学不良反应，发生率为25％。其余不良反应多为1～2级，且无治疗相关死亡。

综上所述，本中心的回顾性资料显示，对于RRMM患者，苯达莫司汀联合泊马度胺及地塞米松方案具有一定的疗效和良好的安全性。尽管目前国内外CD38单抗、XPO1抑制剂、卡非佐米等新药治疗RRMM取得了一定疗效，但对于合并髓外病变的患者，BPD方案仍值得尝试。本研究样本量较小，随访时间较短，BPD方案的长期疗效及安全性还有待更大样本量、更长随访时间的临床研究验证。
